# Diagnosis of hyperprolactinemia in women: A Position Statement from the Brazilian Federation of Gynecology and Obstetrics Associations (Febrasgo) and the Brazilian Society of Endocrinology and Metabolism (SBEM)

**DOI:** 10.20945/2359-4292-2023-0502

**Published:** 2024-04-09

**Authors:** Andrea Glezer, Heraldo Mendes Garmes, Leandro Kasuki, Manoel Martins, Paula Condé Lamparelli Elias, Vania dos Santos Nunes Nogueira, Ana Carolina Japur de Sá Rosa-e-Silva, Gustavo Arantes Rosa Maciel, Cristina Laguna Benetti-Pinto, Andrea Prestes Nácul

**Affiliations:** 1 Universidade de São Paulo Faculdade de Medicina Hospital das Clínicas São Paulo SP Brasil Hospital das Clínicas, Faculdade de Medicina, Universidade de São Paulo, São Paulo, SP, Brasil; 2 Universidade Estadual de Campinas Faculdade de Ciências Médicas Campinas SP Brasil Faculdade de Ciências Médicas, Universidade Estadual de Campinas, Campinas, SP, Brasil; 3 Universidade Federal do Rio de Janeiro Hospital Universitário Clementino Fraga Filho Rio de Janeiro RJ Brasil Hospital Universitário Clementino Fraga Filho, Universidade Federal do Rio de Janeiro, Rio de Janeiro, RJ, Brasil; 4 Universidade Federal do Ceará Núcleo de Pesquisa e Desenvolvimento de Medicamentos Departamento de Medicina Clínica Fortaleza CE Brasil Departamento de Medicina Clínica e Núcleo de Pesquisa e Desenvolvimento de Medicamentos, Universidade Federal do Ceará, Fortaleza, CE, Brasil; 5 Universidade Federal do Ceará Faculdade de Medicina de Ribeirão Preto Hospital das Clínicas Ribeirão Preto SP Brasil Departamento de Clínica Médica, Hospital das Clínicas, Faculdade de Medicina de Ribeirão Preto, Universidade de São Paulo, Ribeirão Preto, SP, Brasil; 6 Universidade Estadual Paulista Faculdade de Medicina de Botucatu Departamento de Clínica Médica Botucatu SP Brasil Departamento de Clínica Médica, Faculdade de Medicina de Botucatu, Universidade Estadual Paulista, Botucatu, SP, Brasil; 7 Universidade de São Paulo Faculdade de Medicina de Ribeirão Preto Departamento de Ginecologia e Obstetrícia Ribeirão Preto SP Brasil Departamento de Ginecologia e Obstetrícia, Faculdade de Medicina de Ribeirão Preto, Universidade de São Paulo, Ribeirão Preto, SP, Brasil; 8 Universidade de São Paulo Faculdade de Medicina Hospital das Clínicas São Paulo SP Brasil Departamento de Obstetrícia e Ginecologia, Disciplina de Ginecologia, Hospital das Clínicas, Faculdade de Medicina, Universidade de São Paulo, São Paulo, SP, Brasil; 9 Universidade Estadual de Campinas Faculdade de Ciências Médicas Departamento de Obstetrícia e Ginecologia Campinas SP Brasil Departamento de Obstetrícia e Ginecologia, Faculdade de Ciências Médicas, Universidade Estadual de Campinas, Unicamp, Campinas, SP, Brasil; 10 Unidade de Reprodução Humana Hospital Femina Grupo Hospitalar Conceição Porto Alegre RS Brasil Unidade de Reprodução Humana, Hospital Femina, Grupo Hospitalar Conceição, Porto Alegre, RS, Brasil

**Keywords:** Prolactinoma, hyperprolactinemia, pituitary tumor, macroprolactin, hook effect

## Abstract

Hyperprolactinemia is a frequent cause of menstrual irregularity, galactorrhea, hypogonadism, and infertility. The most common etiologies of hyperprolactinemia can be classified as physiological, pharmacological, and pathological. Among pathological conditions, it is essential to distinguish prolactinomas from other tumors and pituitary lesions presenting with hyperprolactinemia due to pituitary stalk disconnection. Proper investigation considering clinical data, laboratory tests, and, if necessary, imaging evaluation, is important to identify the correct cause of hyperprolactinemia and manage the patient properly. This position statement by the Brazilian Federation of Gynecology and Obstetrics Associations (Febrasgo) and Brazilian Society of Endocrinology and Metabolism (SBEM) addresses the recommendations for measurement of serum prolactin levels and the investigations of symptomatic and asymptomatic hyperprolactinemia and medication-induced hyperprolactinemia in women.

## KEY POINTS

- Hyperprolactinemia is a cause of menstrual irregularity, galactorrhea, hypogonadism, and infertility.
- Serum prolactin measurement should only be performed in the presence of symptoms compatible with hyperprolactinemia and/or in the presence of a pituitary tumor, even if the diagnosis is incidental. Measurement of prolactin levels is not recommended as a routine evaluation.
- Hyperprolactinemia has several causes. In most cases, it is caused by pregnancy, hypothalamic-pituitary disconnection, or prolactin-secreting pituitary adenomas (prolactinomas), but it can also be secondary to the use of medications.
- Recognizing clinical, laboratory, and imaging findings is essential for diagnosing prolactinomas and their differential diagnoses.

## RECOMMENDATIONS

- Hyperprolactinemia is a condition with diverse etiologies, and its correct identification is essential for proper treatment and monitoring.
- Mild hyperprolactinemia should be confirmed with another prolactin measurement after excluding venipuncture stress.
- Testing for macroprolactin is indicated in patients with asymptomatic hyperprolactinemia.
- If medication-induced hyperprolactinemia is suspected, another serum prolactin measurement is recommended 3 days after discontinuation of the drug, provided withdrawal is feasible. If there are contraindications for discontinuing the drug or persistent doubts regarding the etiology of the hyperprolactinemia, pituitary imaging should be performed.
- Pituitary imaging by magnetic resonance, ideally, or computed tomography if the former is unavailable, should only be obtained after excluding other causes of hyperprolactinemia. When pituitary imaging suggests the presence of a pituitary tumor, it should be evaluated if the size of the lesion and the prolactin levels point to the presumptive diagnosis of prolactinoma.

## BACKGROUND

Increased serum prolactin levels are a common finding in the work-up of patients with complaints of menstrual irregularity and infertility. Considering that a proper evaluation of these patients is essential for their correct management, experts from the Brazilian Society of Endocrinology and Metabolism (SBEM) and the Brazilian Federation of Gynecology and Obstetrics Associations (Febrasgo) prepared this position statement to provide clarification to the medical community on the main points related to the management of hyperprolactinemia.

Hyperprolactinemia is defined by a serum prolactin level above the reference value for the normal population. Normal (or physiological) prolactin levels values are higher in women than men and are generally below 25 ng/mL. Notably, the reference values vary depending on the assay used for measurement.

A single prolactin measurement is sufficient to establish the diagnosis of hyperprolactinemia in most cases. Given the pulsatile secretion of this hormone, a new measurement may sometimes be necessary in patients with mild hyperprolactinemia. Prolactin release stimulation tests (*e.g.,* prolactin quantification after administration of thyrotropin-releasing hormone [TRH]) are not recommended.

Prolactin is the only pituitary hormone under negative tonus, which is mediated by hypothalamic dopamine. It is the main hormone responsible for lactation in women and plays a role in regulating reproductive function by suppressing the gonadotropic axis. In most cases, hyperprolactinemia is caused by pregnancy, hypothalamic-pituitary disconnection, or prolactin-secreting pituitary adenomas (prolactinomas), but may also be secondary to the use of certain medications (Table 1). Regardless of the etiology, hyperprolactinemia can cause hypogonadism, infertility, and galactorrhea. Once hyperprolactinemia has been confirmed, it is important to identify its underlying etiology to ensure proper management.

**Table 1:** Non-tumoral causes of hyperprolactinemia

Physiological	Systemic	Pharmacological
Pregnancy	End-stage chronic kidney disease	Antidepressants, neuroleptics (antipsychotics)
Coitus	Polycystic ovary syndrome	Estrogens (oral contraceptives)
Breastfeeding	Cirrhosis	Antiemetics, antihistamines
Exercise	Chest wall disorders (surgery, shingles, piercing)	Antihypertensives, opioids
Stress	Primary hypothyroidism	Dopamine inhibitors

Adapted from Melmed (2011) (5).

### What is the frequency of hyperprolactinemia in the general population and in subgroups of patients?

Hyperprolactinemia is a cause of amenorrhea in 10%-20% of nonpregnant women (1). In a study of 1,607 patients with clinically treated hyperprolactinemia, the prevalence in men and women was approximately 10 and 30 per 100,000, respectively, with a peak prevalence in women aged between 25-34 years (2). Prolactinomas are the most common functioning pituitary adenomas, occurring at an annual incidence of approximately 30 per 100,000 inhabitants (3). In autopsy studies, the prevalence is much higher, as pituitary adenomas have been identified in up to 11% of the autopsies, with almost half showing positive immunohistochemistry for prolactin (4).

### In which patients should prolactin be measured?

Serum prolactin levels should only be measured in patients with symptoms suggestive of hyperprolactinemia, including galactorrhea, menstrual irregularity, infertility, or decreased libido (Table 2). Additionally, patients undergoing investigation of any hypothalamic-pituitary dysfunction (*e.g.*, pituitary tumors, pituitary stalk lesions) should have serum prolactin measured. However, measuring prolactin as a routine check-up is not recommended. Routine serum prolactin screening in asymptomatic patients may lead to unnecessary costs and interventions. For instance, elevated prolactin level in a patient with pituitary incidentaloma may be attributable to macroprolactinemia.

**Table 2:** Main signs and symptoms of hyperprolactinemia

Infertility
Oligomenorrhea or amenorrhea
Galactorrhea
Hot flushes
Dyspareunia/vaginal dryness
Bone mass reduction
Reduced libido
Signs of tumor compression* (headache, reduced visual field, hypopituitarism)

* Patients with prolactinomas. Adapted from Melmed and cols. (2011) (5).

### What are common laboratory pitfalls in the evaluation of hyperprolactinemia?

The assessment of hyperprolactinemia can be challenging without proper knowledge of the diagnostic pitfalls that can lead to misdiagnosis and inappropriate treatment. Prolactin is usually measured by commercial two-site immunometric assays or sandwich principle immunoassays. Blood samples are usually collected in the morning after 2-3 hours of fasting. If venipuncture poses no challenge, a single blood sample is sufficient to determine the presence of hyperprolactinemia (5,6). The occurrence of mild hyperprolactinemia that is not consistent with the clinical presentation may be due to pulsatile prolactin secretion. Exceptionally, a confirmatory blood draw may be collected in two samples at intervals of 15-20 minutes (7,8).

Two notable conditions regarding the prolactin dosing methodology must be considered, namely, the hook effect and macroprolactinemia (5,6).

#### Hook effect

The hook effect may occur when prolactin levels are measured using sandwich assays. This technique relies on the binding of prolactin to a capture antibody (typically stationary) and a signaling antibody (present freely in the supernatant), forming a “sandwich complex.” After the incubation phase, excess unbound signaling antibodies are washed away, and the sandwich complex signal is read. When prolactin levels are excessively high (generally above 2,000 ng/mL), prolactin binds to both antibodies (capture and signaling), preventing the formation of the sandwich complex and resulting in only moderately elevated concentrations of prolactin (7,8). In cases of large (>3 cm) tumors with prolactin levels 30-120 ng/mL, successive dilutions of the serum are recommended to exclude the hook effect. This is particularly important in patients with pituitary macroadenomas and hyperprolactinemia. In these cases, the prolactin level is the marker distinguishing macroprolactinomas from nonfunctioning pituitary macroadenomas (in which hyperprolactinemia occurs due to pituitary stalk disconnection). Thus, excluding the hook effect in these cases help avoid diagnostic and treatment errors (5,7,8). Importantly, prolactin levels above the method's detection range must also be retested after dilution to obtain the accurate prolactin value.

#### Macroprolactinemia

Macroprolactin is one of the three main forms of circulating prolactin, together with the biologically active 23 kDa monomeric prolactin and the dimeric prolactin (“big prolactin”). Macroprolactin is a macromolecule composed of monomeric prolactin coupled to high molecular weight IgG antibodies (<150 kDa). Although biologically inactive, macroprolactin can be detected by most commercial prolactin assays. Macroprolactin has a high molecular weight and treating the sample with polyethylene glycol (PEG) will cause its precipitation, allowing the quantification of residual monomeric prolactin in the supernatant. The residual prolactin recovered after PEG treatment can be expressed as a percentage of the total prolactin value before PEG treatment. When macroprolactin is the predominant form, residual prolactin after PEG treatment is usually less than 40%. Mild to moderate hyperprolactinemia in an asymptomatic patient should prompt the determination of macroprolactinemia, avoiding unnecessary investigations and interventions (7).

### What are the causes of hyperprolactinemia?

#### Prolactinomas

Prolactinomas, tumors of lactotrophic cells, are the most common pituitary tumors, comprising approximately 50% of all new pituitary adenomas diagnosed annually. The prevalence and incidence of prolactinomas are, respectively, approximately 50 per 100,000 and 3-5 new cases/100,000/year (9). In a Brazilian multicenter study including 1,234 individuals with hyperprolactinemia, prolactinomas accounted for 56.2% of the cases (10). Prolactinomas are classified according to size into microadenomas (<10 mm in diameter) and macroadenomas (≥10 mm). The proportion between microprolactinomas and macroprolactinomas in women has been reported as 8:1, and the peak age of occurrence is around 30 years (11). If no pituitary lesion is identified in patients with hyperprolactinemia, and other causes have been ruled out, the patient is diagnosed with idiopathic hyperprolactinemia. Although prolactinomas are mostly sporadic in cause, approximately 5% of cases may have a familial cause. They may be associated with multiple endocrine neoplasia type 1, isolated familial prolactinomas, and familial pituitary tumors (12). Prolactinoma should be investigated in women with hyperprolactinemia after ruling out physiological or pharmacological causes and chronic diseases that could cause this hormonal change. Prolactinoma should also be investigated in patients with pituitary adenomas, even in the absence of complaints related to hypogonadism. In cases of pituitary tumors impinging on the optical chiasma, neuroophthalmological evaluation should be performed, even in the absence of visual complaints. Additionally, in macroadenomas, a comprehensive evaluation of all pituitary functions is necessary, not limited to the gonadal axis, as other hormonal deficiencies may also occur. Macroprolactinomas should be considered in any patient with neurological or ophthalmological manifestations from mass effect in the sellar region, such as headache or visual field changes with associated hypopituitarism (13). Prolactin should be measured in patients with acromegaly, since they often have concomitant hyperprolactinemia (5). The hyperprolactinemia in these cases may result from pituitary adenomas cosecreting prolactin and growth hormone (GH) (14) or from compression of the pituitary stalk. Patients with acromegaly and concurrent hyperprolactinemia tend to experience an earlier onset of the disease compared with those with purely GH-secreting adenomas, yet exhibit less pronounced physical features of acromegaly (15). Therefore, we recommend screening patients with prolactinoma for acromegaly by measuring insulin-like growth factor type 1 (IGF-1) whenever possible; this should be done at the time of diagnosis and does not need to be repeated during follow-up.

### When prolactinoma is excluded, what other causes of hyperprolactinemia should be investigated?

The main nontumoral cause of hyperprolactinemia is the use of certain medications, a topic that will be covered in the next section. Other nontumoral causes of hyperprolactinemia include physiological conditions, systemic diseases (Table 1), and disorders of the hypothalamic-pituitary region (5,16). The most common physiological causes include pregnancy and breastfeeding, coitus, nipple manipulation, and physical exercise. Except during pregnancy and breastfeeding, in which hyperprolactinemia can reach values above 200 ng/mL, the elevation of prolactin in other situations is mild and rarely leads to galactorrhea or menstrual irregularity (17,18). Systemic diseases can also lead to hyperprolactinemia through different mechanisms. In end-stage chronic kidney disease, decreased clearance of prolactin is the main factor in increasing the levels of this hormone (19). Alterations in the nerve endings of the chest wall result in decreased inhibition by dopamine (20), a phenomenon also observed in liver cirrhosis, where increased estrogen concentrations are present (21). Hyperprolactinemia is found in 20%-40% of patients with primary hypothyroidism and is caused by the elevation of TRH, a lactotroph secretagogue. The elevation in prolactin level appears to be directly related to TSH levels, which should always be measured as part of the differential diagnosis for hyperprolactinemia. With the treatment of hypothyroidism, prolactin levels normalize (22,23). The prevalence of hyperprolactinemia in patients with polycystic ovary syndrome (PCOS) is quite variable. Therefore, hyperprolactinemia associated with PCOS should be a diagnosis of exclusion. Notably, prolactin values above 60-80 ng/mL suggest another underlying cause of hyperprolactinemia that should be actively investigated (24,25). Hypothalamic-pituitary diseases comprise several entities, including neoplastic, granulomatous, infectious and infiltrative diseases, and can cause pituitary stalk disconnection either by sectioning, compressing, or stretching the stalk (Table 3). Consequently, there will be a decrease in the dopaminergic inhibitory tone on prolactin secretion, resulting in hyperprolactinemia. In these cases, the hyperprolactinemia is typically more pronounced with obvious symptoms (galactorrhea, infertility, and hypogonadism) but rarely exceeds 100 ng/mL (26). Therefore, in cases of patients with persistent hyperprolactinemia, when other etiologies having been ruled out, it is recommended to perform imaging examination of the sella turcica, preferably with magnetic resonance imaging (MRI) or computed tomography, when MRI is not available. The differential diagnosis for hyperprolactinemia is of paramount importance, since in many of these etiologies, specific treatment other than with dopaminergic agonists is indicated. Notably, starting dopaminergic agonist treatment for hyperprolactinemia without adequate investigation will delay the diagnosis of the underlying disease with possible disastrous consequences (13,27).

**Table 3:** Conditions of the hypothalamic-pituitary region that can lead to hyperprolactinemia due to stalk disconnection (except for prolactinomas)

Tumors	Infiltrative	Inflammatory	Others
Prolactinomas	Langerhans cell histiocytosis	Lymphocytic hypophysitis	Rathke's pouch cyst
Pituitary macroadenomas (nonfunctioning, secreting, Nelson's syndrome*)	Sarcoidosis	IgG4-related hypophysitis	Section of the pituitary stalk (trauma)
Craniopharyngioma	Tuberculosis	Hypophysitis in granulomatosis with polyangiitis	Internal carotid artery aneurysm
Metastases (breast, lung)			Empty sella
Germ cell tumors			Radiotherapy
Others			Idiopathic

* Nelson's syndrome: growth of corticotropinoma with substantial elevation in serum adrenocorticotropic hormone (ACTH) levels, which may occur after bilateral adrenalectomy. Adapted from Melmed and cols. (2011) (5).

In cases of positive imaging findings, the etiology of hyperprolactinemia can be defined by evaluating the correlation between hyperprolactinemia and the size of the pituitary lesion. Most authors agree that the diagnosis of prolactinoma is suggested in the presence of an image suggestive of pituitary macroadenoma, prolactin levels above 200 ng/mL, and absence of other causes for the hyperprolactinemia, whereas stalk disconnection is suggested if prolactin levels are below 100 ng/mL (5,26).

However, doubt about the etiology of hyperprolactinemia may persist in some cases of pituitary adenoma. Prolactin levels in pituitary stalk disconnection caused by macroadenomas and small prolactinomas may overlap. Pituitary incidentalomas are common, with an estimated global prevalence of 16.7% (28). Therefore, in cases of small, nonfunctioning pituitary adenomas with mildly elevated prolactin levels, the differentiation between microprolactinoma and nonfunctioning incidentaloma is not as clear initially. Only a reduction in tumor size after treatment with dopaminergic agonists can confirm the diagnosis of prolactinoma, as it is known that nonfunctioning microadenomas increase in size in approximately 10%-20% of cases or remain stable (29,30), while microprolactinomas regress in size or disappear after therapy with dopaminergic agonists in the vast majority of the cases. Notably, a surgical series has shown that approximately 17% of pituitary microadenomas considered to be prolactinomas before surgery were not confirmed to be prolactinomas on immunohistochemistry, showing that they were, in fact, nonfunctioning lesions erroneously diagnosed as prolactinomas due to stalk disconnection (31). These data reinforce the importance of monitoring tumor size after prescribing dopaminergic agonists. This monitoring is crucial for distinguishing between prolactinomas and nonfunctioning pituitary lesions increasing prolactin levels due to pituitary stalk disconnection.

### How to manage medication-induced hyperprolactinemia?

Medications are a frequent cause of hyperprolactinemia. Of all causes of hyperprolactinemia, 15%-45% are estimated to be induced by medications (10,32,33). Many medications can cause hyperprolactinemia to varying degrees. Antipsychotics, particularly first-generation ones, are the drugs most frequently associated with hyperprolactinemia. Several other medications with less effect on prolactin levels are currently available (Table 4).

**Table 4:** Medications that cause hyperprolactinemia

**Antipsychotics**		
	Chlorpromazine/thioridazine/levomepromazine	+++
	Haloperidol	+++
	Sulpiride/tiapride	+++
	Risperidone	+++
	Quetiapine	+
	Olanzapine	+
	Pimozide	+
	Clozapine	0
	Aripiprazole	0
**Antidepressants**		
	Clomipramine	+++
	Amitriptyline	+
	Citalopram	±
	Fluvoxamine	±
	Paroxetine	±
	Fluoxetine	CR
	Imipramine	CR
	Bupropion	0
	Nortriptyline	0
	Sertraline	0
	Trazodone	0
**Monoamine oxidase inhibitors**		
	Pargyline	+++
	Clorgyline	+++
	Tranylcypromine	±
**Antihypertensives**		
	Reserpine	++
	Methyldopa	+
	Verapamil	+
	Labetalol	+
**Gastrointestinal agents**		
	Domperidone/metoclopramide	+++
	Cimetidine/ranitidine	+
**Appetite suppressants**		
	Fenfluramine/amphetamines	+
**Opiates and cocaine**		+
**Protease inhibitors**		+
**Estrogen**		+

Abbreviations and symbols:

CR report of isolated cases;

0 no effect;

± minimal increase, but not to levels above normal;

+ increase to levels above normal in a small percentage of patients;

++ increase to levels above normal in 25%-50% of patients;

+++ increase to levels above normal in more than 50% of patients, reaching values > 200 ng/mL. Adapted from Molitch (2008) (34).

The mechanisms by which medications lead to hyperprolactinemia include the inhibition of dopamine by antagonistic action of these substances on dopamine receptors (antipsychotics and metoclopramide) or inhibition of dopamine synthesis (estrogen) (32). Other substances can act on the secretion of factors that alter the tonic suppression of prolactin synthesis, such as serotonin and gamma-aminobutyric acid (GABA). These mechanisms affect the inhibitory control of dopamine. From a practical point of view, in most cases of medication-induced hyperprolactinemia, prolactin levels are below 100 ng/mL (35). However, prolactin levels below 100 ng/mL should not be taken as sole evidence that the cause is only related to medications (35), and establishing a differential diagnosis to distinguish organic causes is one of the first steps in the investigation (4,8). The same manifestations can be caused by medication-induced hyperprolactinemia or prolactin-producing tumors, *i.e.,* menstrual irregularities, galactorrhea, and infertility (4,10). Therefore, adopting some strategies may help differentiate between these two causes, for example, remeasuring prolactin levels after temporarily stopping or changing the medication to observe the impact on prolactin level or, when this is not possible, investigate using imaging methods. Notably, the withdraw or change of medication should always be done in conjunction with the psychiatrist or prescriber of the medication causing the increase in prolactin level. An additional suggestion is to investigate using imaging methods those patients with prolactin levels greater than 100 ng/mL who use medications known to increase prolactin (35). Sometimes, after excluding tumor-related causes (especially macroadenomas), the medication causing increased prolactin levels cannot be changed. In such cases, for women with a uterus, combined estrogen and progestogen can be considered to minimize the consequences of hypoestrogenism. When contraception is necessary, combined hormonal contraceptives may be considered.

### What changes regarding the diagnosis of hyperprolactinemia after menopause?

Hyperprolactinemia is usually diagnosed in young women during reproductive years. Currently, there is little evidence on how hormonal changes resulting from menopause interfere with prolactin production. Since postmenopausal women have cessation of menstrual periods, they do not experience the classic manifestation of menstrual irregularity due to hyperprolactinemia. This absence of menstrual symptoms can delay the diagnosis of pituitary adenomas, leaving the true incidence of prolactinomas at this stage of life unknown. Microadenomas are rarely diagnosed after menopause. The most frequently diagnosed lesions in this age group are larger tumors that are often investigated due to the presence of compressive symptoms (visual changes and headache) resulting from mass effect with eventual parasellar extension. A multicenter study including 14 women with macroadenomas diagnosed after menopause has reported that 6 of them had visual changes and only 3 had galactorrhea (36). These patients had relatively high initial prolactin levels and responded well to dopaminergic agonists despite the late diagnosis. Still, there is little data in the literature on prolactinomas diagnosed during menopause (37), and 92% of the cases described have been macroprolactinomas.

**Figure 1:**
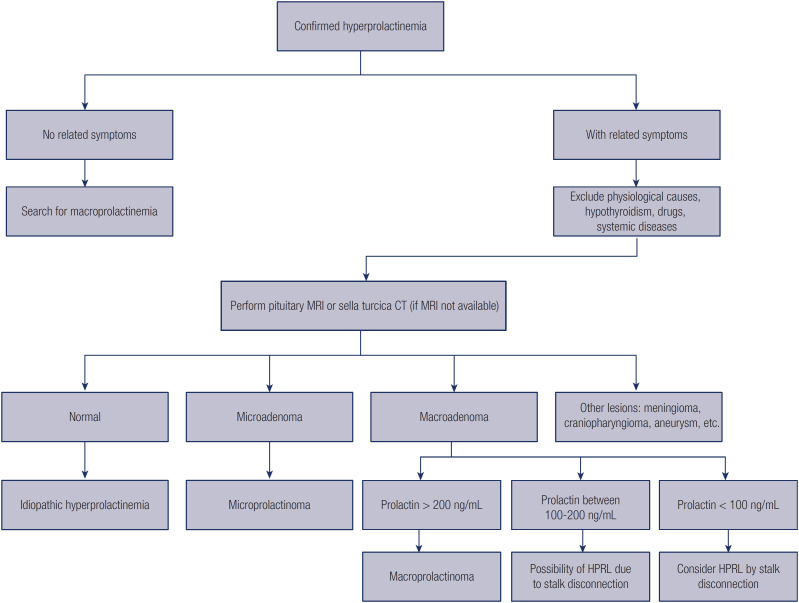
Flow chart for the diagnostic assessment of hyperprolactinemia.

Abbreviations: CT, computed tomography; HPRL, hyperprolactinemia; MRI, magnetic resonance imaging.

In summary, we suggest the following flow chart for diagnosing hyperprolactinemia (Figure 1).

In conclusion, this position statement was prepared jointly by the Department of Neuroendocrinology of the Brazilian Society of Endocrinology and Metabolism (SBEM) and the National Specialized Commission on Gynecological Endocrinology of the Brazilian Federation of Gynecology and Obstetrics Associations (FEBRASGO). The aim of this document is to provide updated information to assist gynecologists, endocrinologists, and primary care physicians in diagnosing hyperprolactinemia in women.
